# RETRACTION: Effect of Thoracoscopic and Thoracotomy on Postoperative Wound Complications in Patients With Lung Cancer: A Meta‐Analysis

**DOI:** 10.1111/iwj.70381

**Published:** 2025-03-21

## Abstract

**RETRACTION:** QiuB. , HanJ. , and ZhaoJ. , “Effect of Thoracoscopic and Thoracotomy on Postoperative Wound Complications in Patients with Lung Cancer: A Meta‐Analysis,” International Wound Journal 20, no. 10 (2023): 4217–4226, 10.1111/iwj.14322.37596788 PMC10681477

The above article, published online on 18 August 2023, in Wiley Online Library (http://onlinelibrary.wiley.com/), has been retracted by agreement between the journal Editor in Chief, Professor Keith Harding; and John Wiley & Sons Ltd. Following an investigation by the publisher, all parties have concluded that this article was accepted solely on the basis of a compromised peer review process. The editors have therefore decided to retract the article. The authors did not respond to our notice regarding the retraction.

**RETRACTION:** B. Qiu , J. Han , and J. Zhao , “Effect of Thoracoscopic and Thoracotomy on Postoperative Wound Complications in Patients with Lung Cancer: A Meta‐Analysis,” International Wound Journal 20, no. 10 (2023): 4217–4226, 10.1111/iwj.14322.37596788 PMC10681477

The above article, published online on 18 August 2023, in Wiley Online Library (http://onlinelibrary.wiley.com/), has been retracted by agreement between the journal Editor in Chief, Professor Keith Harding; and John Wiley & Sons Ltd. Following an investigation by the publisher, all parties have concluded that this article was accepted solely on the basis of a compromised peer review process. The editors have therefore decided to retract the article. The authors did not respond to our notice regarding the retraction.

